# Rehabilitation Techniques Before and After Total Knee Arthroplasty for a Better Quality of Life

**DOI:** 10.7759/cureus.54877

**Published:** 2024-02-25

**Authors:** Sushmita Dutta, Ratnakar Ambade, Dhanashree Wankhade, Palak Agrawal

**Affiliations:** 1 Orthopaedics, Jawaharlal Nehru Medical College, Datta Meghe Institute of Higher Education & Research, Wardha, IND

**Keywords:** perturbation drill, prehabilitation, rheumatoid arthritis, osteoarthritis, strengthening exercises

## Abstract

The most important gold standard treatment following advanced knee osteoarthritis is total knee arthroplasty. Following surgery of total knee replacement, the majority of patients report decreased pain and successful long-term results, but recovery is unpredictable, and most patients continue to exhibit muscle weakness in their lower limbs and functional limitations in comparison to similarly aged control individuals. The goal of this review article was to systematically review different articles containing controlled and randomized studies to find out the effectiveness of outpatient care postoperatively on short- and long-term functional recovery. The purpose of this review article is to investigate the possible advantages of pre- and postoperative rehabilitation as well as the value of exercise regimen recommendations following total knee replacement. The following interventions after total knee arthroplasty are discussed in this review article: preoperative education and exercises, continuous passive movement, strengthening interventions, aquatic therapy, balanced training, tourniquet exposure, use of alignment and implants, role of apps in phones and different wearable devices, influence of postoperative protocols, knee bracing, neuromuscular electrical stimulation, and clinical environment. Strengthening and intense functional exercises for patients above 45 years of age, in land or water programs like aquatic activities, with the increasing intensity of the exercises in accordance with the patient's progress, should be included in the best outpatient physical therapy protocols. Because these exercises are so precisely personalized, the best long-term effects after surgery may come from outpatient physiotherapy performed in a clinical setting under the supervision of a registered physiotherapist or medical professional. This review article also includes the change in the quality and well-being of a patient's life who has undergone total knee arthroplasty and practiced the rehabilitation techniques.

## Introduction and background

The most prevalent conditions that result in knee joint disease are arthritis and rheumatoid arthritis. These situations can cause nerve and muscle pain, loss of movement, and other symptoms that keep patients from going about their everyday lives and working [1]. For patients with severe osteoarthritis of the knee, total knee arthroplasty is an effective treatment that can effectively rectify joint deformities, lessen discomfort, enhance knee function, and enhance patients' quality of life [2]. An established standard method to relieve the symptoms of advanced knee osteoarthritis is total knee arthroplasty. Over the past 20 years, there has been a steady rise in the annual incidence of total knee arthroplasty over the world [3]. After a total knee replacement, the patient's range of motion is restricted and limited because of weakened muscles. One month after a total knee replacement, for example, muscle function was shown to be lowered by 20%-25% [4], but one year later, it was still lower than in healthy adults, with reports of 18% slower walking speed and 51% slower stair climbing speed [5]. Furthermore, discomfort, edema, and hemorrhage following surgery restrict the range of motion of the knee joint [6]. As a result, only 67% of patients will regain full functioning abilities [7]. In addition, even though rehabilitation was started within 48 hours following surgery, the quadriceps muscle's strength decreased by 30.7% right away and by 50%-60% after a month [8].

Therefore, postoperative rehabilitation programs are crucial because they can enhance patient function, results, and mobility following total knee arthroplasty. These programs include fitness elements that involve motion- and strength-strengthening exercises for the muscles and joints, as well as walking exercises and exercises for function, endurance, and balance. To restore the normal range of motion that helps restore muscle strength by allowing full muscular contraction, the full range of motion must be regained. Walking and functional exercises enhance blood flow and restore the capacity to carry out daily activities, including standing, sitting, and climbing stairs [9]. Exercises for postoperative rehabilitation can pull and stretch muscles; build muscle strength; enhance local blood circulation; avoid issues such as nerve root adhesion, knee stiffness, and thrombosis; and hasten the recovery of the knee joint [10]. The majority of recent research in this area, however, has concentrated on postoperative functional exercise, paying little attention to preoperative exercise intervention. In actuality, preoperative exercise for limb function is just as crucial as postoperative exercise for limb function after total knee arthroplasty. Preoperative functional exercise can help the patient gain more strength, adjust to postoperative functional exercise more easily, and speed up the recovery of walking function, in addition to strengthening the muscles around the injured joints [11].

## Review

Methods

**Figure 1:**
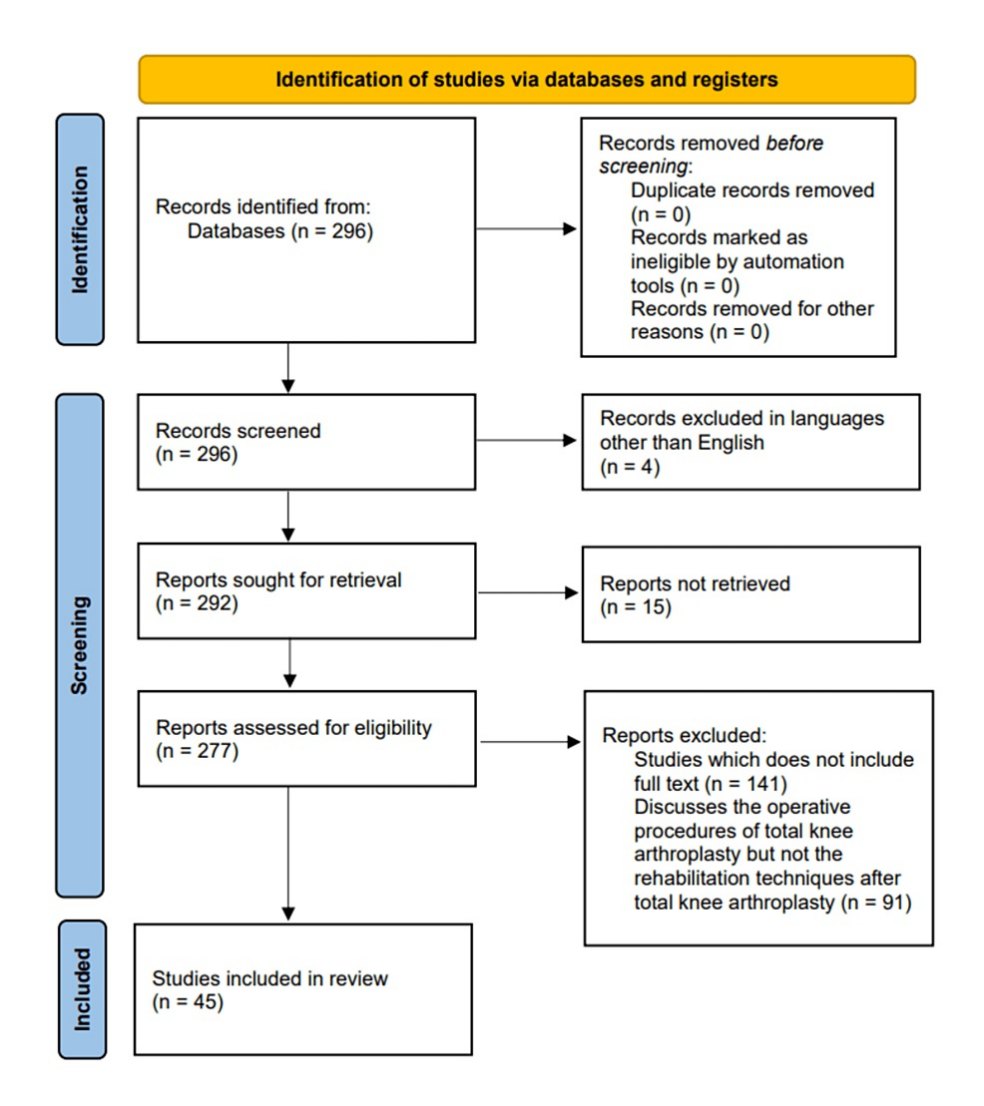
PRISMA flow diagram for the process of selection PRISMA: Preferred Reporting Items for Systematic Reviews and Meta-Analyses.

A comprehensive study was done about different research works, which included randomized control trials (RCTs), which were analyzed through meta-analysis and risk of bias assessment. Reliable computer databases like PubMed and Google Scholar, which were published in the last 10 years, were searched for appropriate data. All searches included full-text review articles that were published and included key terms such as "total knee arthroplasty," "rehabilitation techniques," "physiotherapy," "osteoarthritis," and "prehabilitation," which were used interchangeably and in combination. All articles that discussed the rehabilitation techniques after total knee arthroplasty were included and that only discussed the operative procedures were excluded. Out of 296 articles identified, 45 were deemed relevant and were included. The Preferred Reporting Items for Systematic Reviews and Meta-Analyses (PRISMA) flowchart for the literature search is depicted in Figure 1.

Etiology

Total knee arthroplasty involves removing the damaged or deteriorated articular surfaces of the knee and replacing them with components made of metal and polyethylene. For more than 95% of total knee arthroplasty operations, the disease or degeneration is caused by the breakdown of the joint cartilage by osteoarthritis, rheumatoid arthritis, posttraumatic degenerative joint disease, or other pathologic diseases [12].

Preoperative education

The results of total knee arthroplasty will be influenced by both curable or modifiable risk factors and non-modifiable risk factors. Understanding and appreciating the risk factors might aid with prognosis and care planning. Prior to surgery, patients getting a total knee arthroplasty should receive education from the physical therapists or other team members. Patient expectations during hospitalization, variables influencing discharge planning and disposition, a postoperative rehabilitation program, safe transferring procedures, the use of assistive equipment, and fall avoidance should, at the very least, be covered in this instruction [12].

Preoperative exercises

Prehabilitation raises physical activity levels both before and after total knee replacement. Prior to total knee arthroplasty, patients' pain and degree of physical activity in everyday activities improved after an eight-week exercise and education program. The program had a good impact on postsurgical satisfaction since the patients felt well-prepared [13]. Overall, it implies that prior to knee replacement, prehabilitation may enhance patients' pain and function. However, when provided as part of routine care prior to knee replacement, these gains might not be clinically significant. Preoperative instruction influenced patients' expectations favorably and could lead to greater satisfaction. Group exercise's social component may have a good impact on compliance and engagement [13].

Use of continuous passive movement (CPM) device for mobilization

A computerized device that gently moves the knee through a predetermined range of motion is used to achieve continuous passive movement (CPM). With the addition of CPM, it is thought that the healing of soft tissues is enhanced, complications are reduced, and length of stay (LOS) at the hospital is decreased [14].

The benefits of using CPM are that the results for function-related outcomes were not significant, and the hospital duration of stay results were not statistically significant. The risks, damages, and/or costs are that the use of CPM may cause bed rest to last longer, the use of it is inconvenient, and even though expenditures were not disclosed in the studies, an associated expense is anticipated.

Organizations have the right to assess the use of CPM following total knee arthroplasty and discourage it unless there are clear difficulties with the surgery [12]. In the early postoperative stage, the inclusion of CPM did considerably enhance knee flexion, although the improvement might not have had clinical significance. Additionally, there were no significant effects on long-term clinical and functional outcomes following total knee arthroplasty, suggesting that routine CPM administration in the aforementioned context may be discontinued [2].

Strengthening interventions

A number of neuromotor changes that take place as people age cause skeletal muscle weakening and decreased strength. Muscular power and strength are reduced by at least 24% in total knee arthroplasty patients compared to the normal limb [15]. According to researchers, more strenuous rehabilitation regimens could be able to address these impairments. Recent studies have focused on rehabilitation tactics that take movement velocity, a power component, into account. High-velocity (HV) training is hypothesized to increase functional mobility due to its preferential stimulation of type 2 muscle fibers [16]. A muscle contraction must be performed as swiftly as possible, or in less than a second, to qualify as this type of exercise. A low-velocity (LV) muscular contraction takes place in two seconds or longer as opposed to an HV. Evidence suggests that HV workouts help reduce quadriceps impairment and enhance static and dynamic balance [17].

Using a methodology for gradual strengthening following total knee replacement resulted in significantly better one-year outcomes for strength of quadriceps (+21%), Timed Up and Go (TUG) (-44% and -24%, respectively), Stair Climbing Test (SCT) times, and distance walked in the Six-Minute Walk (6MW) test (+15%) in comparison to an RCT integrated cohort that underwent standard rehabilitation centered on functional training [4]. The quadriceps strength, flexion range of motion (ROM), and TUG time significantly increased following a strengthening program for four weeks using a vibration platform for the whole body [18].

Quadriceps femoris muscle strength is a crucial factor in determining physical function following total knee replacement. After total knee replacement, quadriceps weakness, which is frequently present in the osteoarthritic limb, gets worse. Even while some quadriceps strength is recovered, it may take more than two years to return to preoperative levels. It is also unknown whether quadriceps strength in the operated limb ever reaches that of the contralateral limb that is disease-free or the quadriceps strength of healthy controls. Studies indicate that "prehabilitation" may be combined with neuromuscular electrical stimulation, progressive resistance training, and quadriceps volitional force production. To ascertain efficacy, well-designed, controlled research is required. Orthopedic surgeons and rehabilitation professionals prioritize reducing quadriceps weakness due to the high prevalence of knee osteoarthritis and total knee arthroplasty [19].

Aquatic therapy

Exercise in warm water, according to practitioners of water-based rehabilitation procedures, may lessen joint stress and help the person strengthen their lower extremities by leveraging water resistance and buoyancy to their advantage. There have been few comparative efficacy and cost-effectiveness studies evaluating the role of aquatic therapy in a population who underwent total knee arthroplasty, and water-based rehabilitation may raise the cost per visit. Early on, following total knee arthroplasty, when pain or muscular limitations make it difficult to conduct resistance exercises in weight-bearing positions, applying the concepts of buoyancy may be the most beneficial. Water-based therapy can be started after six days of total knee arthroplasty, provided the wound is covered using a waterproof adhesive covering [20].

Balanced training

Patients who have total knee arthroplasty and prolonged muscle weakness have a serious impairment in their balance. Patients who have had total knee arthroplasty are more likely to fall and get other orthopedic injuries [21,22]. Following total knee arthroplasty, physical therapy should focus on treating balance issues. With the purpose of enhancing patient stimulation, compliance, and pleasure with therapy, newer interactive technologies have lately been implemented in rehabilitation sessions [23]. The usefulness of including particular balance drills (agility and perturbation drills) in an intensive functional rehabilitation plan was examined in two trials with comparable methodologies. It was discovered that patients who were randomly assigned to receive balance training for one and a half months had faster speed while walking, and in a unilateral balance test using a single leg stance, they performed better than subjects who were randomized only to receive the rigorous functional rehabilitation regimen. In this study, the 30-second chair rise test showed similar improvements in both groups. However, no tests of significance were run in this investigation, and only confidence intervals were given [24].

Tourniquet exposure

Although it had no long-term impact, avoiding employing a tourniquet sped up the recovery of extensor mechanism lockout [25]. There was no difference after one year, although others observed a substantial difference in function within the first six months after total knee replacement compared to applying a tourniquet [26]. The decrease in quadriceps muscle volume and strength associated with the use of tourniquets can be used to explain this finding [27].

Use of alignment

Regardless of the postoperative alignment, recent investigations on kinematic alignment found that total knee arthroplasty performed by the designers had good functional outcomes along with a follow-up of six years [28]. However, a randomized controlled trial comparing mechanical alignment to kinematic alignment at one year postoperatively found no functional changes [29].

A recent survey found that computer-assisted surgery (CAS) produces better immediate functional outcomes [30]. It is challenging to interpret this finding as this improvement was unrelated to better implant location [31]. Patient-specific instruments (PSIs) advantages have not been established [32,33]. Their usefulness may be questioned because of their high cost, delay in design and manufacturing, and lack of proof that PSI is superior to CAS or other types of instrumentation [34].

Design of implant

It is debatable which implant kind is better than the other [35]. There is no discernible difference between the various implant kinds (type of posterior stabilization, posterior cruciate sacrifice or not, mobile or fixed bearing, etc.) in terms of the patient-reported outcome measures and quality of life [36]. Some authors claim that "high-flex" implants increase postoperative flexion and are only used in younger patients or those with little flexion preoperatively [37]. However, there is no proof that its design facilitates a quicker return to mobility [38].

Up to three years following the total knee arthroplasty, improved design of implants (tibial baseplate, posterior condyles, and trochlear groove) and a wider range of sizes offered by contemporary models of the implant have a good impact on patient satisfaction. They also avoid implant overhang, which is known to have detrimental effects [39]. A recent study showed no differences between cementless and cemented implants in terms of implant fixation, with the exception of the Knee Society Score (KSS knee score) after two years of surgery, and there was no impact on patient's contentment or quality of life over time [40].

The role of commercially available smartphone apps and wearable devices

Commercially accessible wearables and smartphone apps have the potential to supplement or completely replace conventional rehabilitation regimens by effectively tracking physical activity and enhancing patient participation after total knee arthroplasty. Intervention components, including step objectives, app-based patient interaction platforms, and patient-specific rehabilitation benchmarks, may help programs work better. Future studies should, however, concentrate on the economics of adoption, long-term effects, and compliance and accuracy optimization when employing these devices [41].

Influence of the postoperative protocols

No matter what postoperative rehabilitation strategy is employed, studies consistently demonstrate that quality of life increases after total knee arthroplasty. Based on published studies, it is difficult to evaluate the impact of rehabilitation on this finding. It is specifically challenging because care after surgery differs from nation to nation and perhaps even within a single nation or region. Therefore, even though private centers are more common or even utilized entirely for social and economic reasons, it is difficult to evaluate the relative contributions of rehabilitation in specialist centers to that of private centers [42]. The various pain management techniques after total knee arthroplasty are depicted in Table 1 [43].

**Table 1:** Management of pain after total knee arthroplasty Lavand'homme et al. [43].

Methods to reduce pain
Paracetamol
Nonsteroidal anti-inflammatory drugs, including cyclo-oxygenase-2-specific inhibitors
Glucocorticoids
Gabapentinoids like Gabapentin and Pregabalin
Systemic ketamine
Systemic alpha-2 adrenergic agonists
Intrathecal morphine
Epidural analgesia
Local infiltration analgesia
Adductor canal block
Sciatic nerve block
Femoral nerve block

Knee bracing

Dynamic posterior cruciate ligament (PCL) knee braces are the most current advancement in functional knee bracing. They counteract tibial posterior translation by applying a forward pressure on the posterior proximal tibia to lessen the posterior lag. This represents the biggest advancement in functional knee bracing. An untreated PCL injury can result in long-term instability and early joint aging. For conventional management of injury to the PCL, dynamic PCL braces offer a substantial new therapeutic adjunct [44].

Neuromuscular electrical stimulation (NMES)

For patients who have undergone total knee arthroplasty, physical therapists should use neuromuscular electrical stimulation (NMES) to enhance quadriceps strength, gait performance, performance-based outcomes, and patient-reported results [12]. From two to 52 weeks following a total knee replacement, there is an improvement in the maximum voluntary isometric contractions of the quadriceps and hamstrings. Additionally, there has been improvement in patient-reported outcomes, stair-climbing performance, and walking [12].

Clinical environment

It is ideal for performing outpatient physical therapy in a clinical setting because the physical therapist may closely track the patient's development and adjust the intervention as the patient's functional status changes [45]. Physical therapy performed outside of a hospital is more expensive than exercises done at home and necessitates patients to go to the facility, which may be challenging for an old age population. The question of whether supervised outpatient rehabilitation is better than non-standardized treatment, telerehabilitation (where the patient is monitored remotely by a therapist), and/or rehabilitation at home (with monitoring call) is, therefore, crucial [45]. A summary of all the studies included in this review is given in Table 2.

**Table 2:** Studies included in the review CPM: continuous passive movement.

Authors	Year	Findings
Gränicher et al. [1]	2020	Preoperative physiotherapy has little benefit on total knee arthroplasty.
Wirries et al. [2]	2020	CPM significantly improved knee flexion.
Yelin et al. [3]	2016	There is an increased number of patients reporting for musculoskeletal diseases.
Petterson et al. [4]	2009	There are no adverse events following exercises after total knee arthroplasty.
Bade et al. [5]	2010	After six months, the patients were back to their preoperative status.
Ranawat et al. [6]	2003	Few patients will face difficulty to restore normal life after total knee arthroplasty rehabilitation.
Franklin et al. [7]	2008	Total knee arthroplasty effectively relieves pain due to arthritis.
Mizner et al. [8]	2005	Muscle activation is necessary after total knee arthroplasty.
Chehuen et al. [9]	2017	Walking training also improved cardiovascular function.
Franz et al. [10]	2018	Use of tourniquets can also lead to muscle atrophy.
Liao et al. [11]	2019	Longer duration of CPM use gives a better effect.
Jette et al. [12]	2020	Physiotherapy should start within 24 hours of surgery.
Clode et al. [13]	2018	Prehabilitation improves the patient’s pain before total knee arthroplasty.
Mistry et al. [14]	2016	Early initiation of exercise therapy helps to heal faster.
Walsh et al. [15]	1998	Severe impairments cannot be treated completely.
Doerfler et al. [16]	2016	High-velocity quadriceps exercises are a very effective way to heal after total knee arthroplasty.
Orr et al. [17]	2006	In older persons, power exercise improves balance because it causes a delayed contraction and initially lowers muscle power.
Johnson et al. [18]	2010	An alternate strengthening program for patients recovering from complete knee replacements is whole-body vibration.
Saleh et al. [19]	2010	Neuromuscular electrical stimulation improves the strength of the quadriceps.
Liebs et al. [20]	2012	When treating osteoarthritis in the knee, the effect size of early aquatic therapy following total knee arthroplasty was comparable to that of nonsteroidal anti-inflammatory medication therapy.
Matsumoto et al. [21]	2012	Exercise therapy helps patients with limited knee flexion.
Swinkels et al. [22]	2009	Fall risk assessment should be done.
Fung et al. [23]	2012	Wii Fit may be used in conjunction with physical therapy for outpatients recovering from total knee replacement surgery.
Piva et al. [24]	2010	There is a higher degree of improvement with balanced training.
Jiang et al. [25]	2015	The patients may benefit from using a tourniquet after total knee arthroplasty.
Guler et al. [26]	2016	In total knee arthroplasty, the use of tourniquets reduces the volume of the thigh and quadriceps muscles and postoperatively postpones the restoration of knee function.
Dennis et al. [27]	2016	There are both advantages and disadvantages of using a tourniquet after total knee arthroplasty.
Howell et al. [28]	2013	Regardless of the alignment category, kinematically aligned total knee arthroplasty restores function without catastrophic failure.
Waterson et al. [29]	2016	Mechanical alignment gives the same result as kinematical alignment.
Rebal et al. [30]	2014	More precise alignment is provided by computer navigation in total knee arthroplasty.
Roberts et al. [31]	2015	Computer-assisted navigation is proving very helpful in total knee arthroplasty.
Sassoon et al. [32]	2015	Patient-specific instrumentation requires lesser surgical trays.
Abdel et al. [33]	2014	Following a total knee replacement, there is no discernible benefit in terms of early functional or gait results from patient-specific instrumentation.
Yan et al. [34]	2015	Routine use of patient-specific instrumentation is not advised due to extra expenditure.
Tille et al. [35]	2024	The cruciate-retaining implant is beneficial.
Nunley et al. [36]	2015	Utilizing more modern implant designs did not result in higher patient satisfaction.
Li et al. [37]	2015	Newer prosthesis provided a greater range of motion.
Kim et al. [38]	2016	High flexion prosthesis provided a better range of motion.
Hamilton et al. [39]	2015	The patient outcome is better with using a prosthesis.
Fricka et al. [40]	2015	It is crucial to closely monitor radiolucencies and to follow up on them.
Constantinescu et al. [41]	2022	Wearable technology and commercially available smartphone apps are potentially useful.
Canovas and Dagneaux [42]	2018	Psychological support is also necessary after surgery.
Lavand’homme et al. [43]	2022	Pain relief treatment is necessary after total knee arthroplasty.
Hewlett and Kenney [44]	2019	Newer technologies like knee bracing are useful modalities.
Pozzi et al. [45]	2013	Postoperative physical exercise is very useful after total knee arthroplasty.

## Conclusions

The objective of this review article was to investigate the possible advantages of pre- and postoperative rehabilitation techniques as well as the value of exercise regimen recommendations following total knee replacement. According to the available research, I conclude that prehabilitation (exercise/physiotherapy programs in the months before surgery) has a minimal impact on pain and function in patients undergoing joint replacement and was not sustained over time. Most metrics of patient recovery, quality of life, duration of stay, and expenses did not show clinically significant (or statistically significant) differences as a result of prehabilitation. Future studies must have enough power to measure clinically important outcomes.

Following total knee arthroplasty, postoperative rehabilitation helps patients heal faster and live better. Continuous passive motion and inpatient rehabilitation could not offer the patient or healthcare system any further advantages. Early rehabilitation, telerehabilitation, outpatient therapy, high-intensity exercise, and high-velocity exercise, however, might be effective rehabilitation techniques. Balance control, neuromuscular electrical stimulation, and weight-bearing biofeedback also seem to be helpful additions to traditional rehabilitation.
